# Influenza B virus infection complicated by life-threatening pericarditis: a unique case-report and literature review

**DOI:** 10.1186/s12879-018-3606-7

**Published:** 2019-01-10

**Authors:** Silvia Spoto, Emanuele Valeriani, Luciana Locorriere, Giuseppina Beretta Anguissola, Angelo Lauria Pantano, Francesca Terracciani, Elisabetta Riva, Massimo Ciccozzi, Sebastiano Costantino, Silvia Angeletti

**Affiliations:** 1 Internal Medicine Department, University Campus Bio-Medico of Rome, Italy, Via Alvaro del Portillo, 200 Rome, Italy; 2 Internal Medicine Department, University G. D’Annunzio, Via dei Vestini, 31, Chieti, Italy; 3 Unit of Virology, University Campus Bio-Medico of Rome, Via Alvaro del Portillo, 200 Rome, Italy; 4 Unit of Medical Statistic and Molecular Epidemiology, University Campus Bio-Medico of Rome, Via Alvaro del Portillo, 200 Rome, Italy; 5 Unit of Clinical Laboratory Science, University Campus Bio-Medico of Rome, Via Alvaro del Portillo, 200 Rome, Italy

**Keywords:** Type B influenza virus, Pericarditis, Pericardial effusion, Vaccination, Down syndrome, Congenital heart disease with pulmonary hyper flow

## Abstract

**Background:**

Acute pericarditis may occur frequently after viral infections. To our knowledge, influenza B virus infection complicated by pericarditis without myocardial involvement has never been reported. We report the first case of life-threatening pericarditis caused by influenza B virus infection.

**Case presentation:**

A 48-years-old woman with trisomy 21 and ostium primum atrial septal defect was transferred from Cardiology to our Internal Medicine Department for severe pericardial effusion unresponsive to ibuprofen and colchicine. Based on the recent patient history of flu-like syndrome, and presence of pleuro-pericardial effusion, a viral etiology was suspected. Laboratory evaluation and molecular assay of tracheal aspirate identified influenza B virus. Therefore, the ongoing metilprednisolone and colchicine therapy was implemented with oseltamivir with progressive patient improvement and no evidence of pericardial effusion recurrence during follow-up.

**Conclusions:**

Especially in autumn and winter periods, clinicians should include Influenza B virus infection on differential diagnosis of pericarditis with large pericardial effusion.

## Background

Acute pericarditis is an inflammatory condition involving pericardium and is responsible for 0.2% of all cardiovascular hospitalization [1]. Almost the whole acute pericarditis (80–90%) are idiopathic in developed countries, suggesting a viral etiology, roughly 70–90% are self-limiting while only 5% are therapy-resistant [2, 3]. Among viral pathogens enteroviruses, herperviruses, parvovirus B19, cytomegalovirus, H1N1, parainfluenza, varicella zoster, HIV, hepatitis B and C are listed; bacterial, fungal, and parasitic infections are rare [4, 5]. Neoplastic, systemic inflammatory syndromes-related, tuberculosis, and purulent pericarditis occurred respectively in 5–10%, 2–27%, 4%, and < 1% of cases [5]. Diagnosis of pericarditis is made by the presence of at least 2 of the following criteria: acute chest or pleuritic pain, pericardial friction rubs, electrocardiogram (ECG)-specific alterations, and evidence of new or worsening pericardial effusion. Among additional criteria, C-reactive protein (CRP) elevation, present in 75% of cases [6], and evidence of pericardial inflammation on computed tomography (CT) or magnetic resonance image are included.

To date, no case of Influenza B virus infection-related pericarditis, without myocardial involvement, has been described. We report this first case of life-threatening pericarditis due to Influenza B virus infection.

### Case presentation

**Fig. 1:**
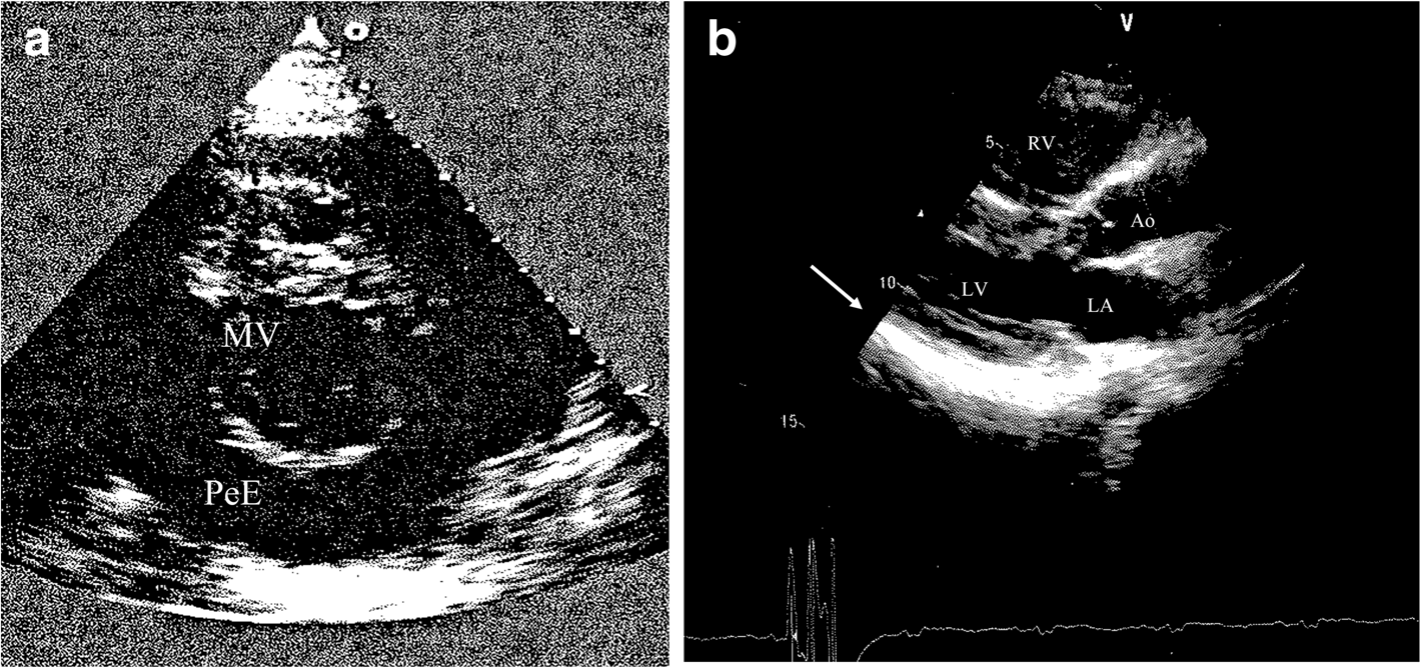
**a** Parasternal short-axis view showing large pericardial effusion (30 mm): > 20 mm “eco-free space” on echocardiogram is indicative of large pericardial effusion. MV: mitral valve; PeE: pericardial effusion. **b** Mild pericardial detachment on right atrium (white arrow) without sign of compression on 1-month echocardiographic follow-up (parasternal long-axis view). Ao: aorta; LA: left atrium; LV: left ventricle; RV: right ventricle

A 48-years-old woman with trisomy 21 and history of ostium primum atrial septal defect and hypothyroidism, on effective replacement therapy, was transferred from the Cardiology of another hospital to our Internal Medicine Department because of dyspnea with acute and worsening respiratory failure. She had been in her usual health until 2 months before admission, when a flu-like syndrome occurred in November. After 15 days, during an admission in other hospital for syncope with sphincter incontinence, a mild pericardial effusion (7 mm) was diagnosed and treated with ibuprofen 600 mg every 8 h and colchicine 0.5 mg twice day. Two weeks later, due to worsening of dyspnea and appearance of diarrhea, therapy had to be suspended. Trans-thoracic echocardiogram showed a diffuse increase in pericardial effusion (30 mm) without inspiratory collapse of the inferior vena cava (Fig. 1a). Chest CT confirmed massive pericardial effusion and highlighted bilateral basal and left upper lobe pleural effusion with atelectasis. Therefore, a metilprednisolone 60 mg/day (1 mg/Kg) and furosemide 40 mg/day therapy was started.

**Fig. 2:**
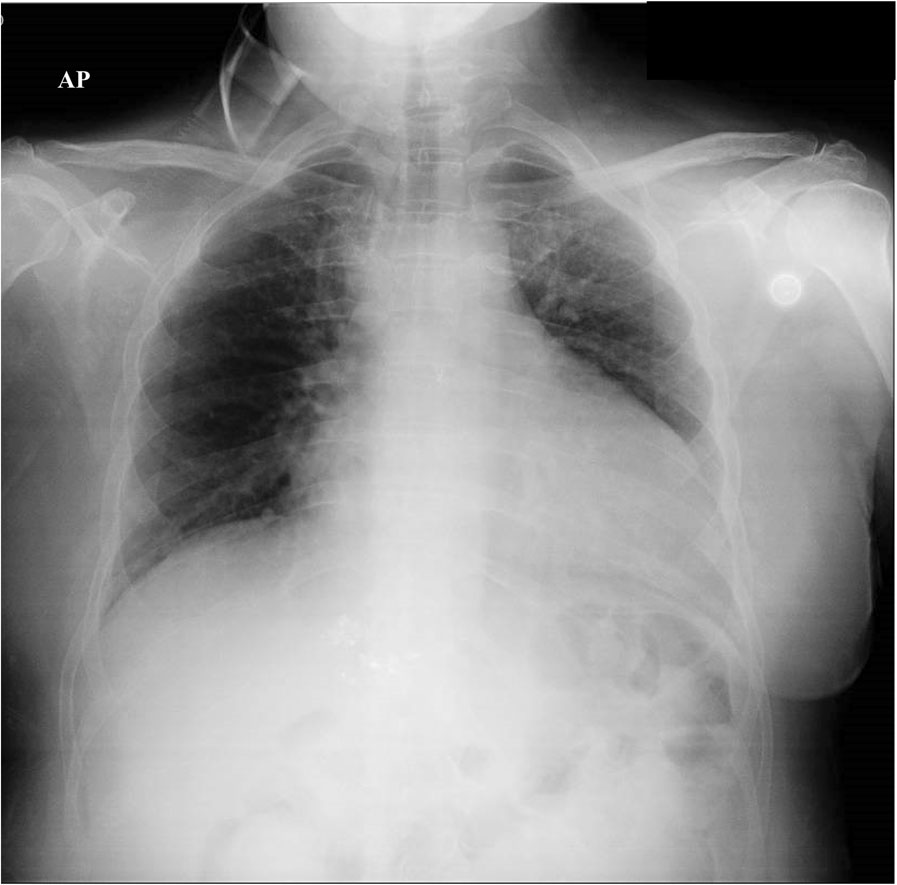
Widespread bronchial wall thickening and enlargement of the cardiac silhouette on chest X-ray from large pericardial effusion

On admission, blood pressure was 110/70 mmHg, heart rate 75 beats per minute, respiratory rate 16 breaths per minute, body temperature 99 °F, and oxygen saturation 86% while the patient was breathing ambient air. Cardiac examination revealed muffled heart sounds and a 3/6 ejection murmur on aortic area. Pulmonary evaluation revealed a stony dull percussion with diminished vesicular breath sounds on basal region bilaterally and widespread rhonchi. The total leukocyte count was 8400/μL (neutrophils 93% and lymphocytes 15%), erythrocyte sedimentation rate (ESR) 120 mm/h, CRP 16 mg/L, procalcitonin 0.05 ng/mL, MR-pro-adrenomedullin 0.96 nmol/L, ferritin 3799 ng/mL, NT-proBNP 254 pg/mL, and TSH 0.33 mcU/L; ANA, anti-dsDNA, ENA, c-ANCA and p-ANCA were in the normal range. Arterial blood gas analysis revealed severe hypoxemia with respiratory and metabolic alkalosis (pH 7.48, pO_2_ 47.3 mmHg, pCO_2_ 36.1 mmHg, HCO^3−^ 26.6 mmol/l). ECG highlighted incomplete right bundle branch block, while trans-thoracic echocardiogram showed large circumferential pericardial effusion (25, 23, and 30 mm respectively on apical, posterior, and lateral ventricular walls, 20 mm on right ventricular wall) without right ventricular compression; grade 2 diastolic dysfunction; mild left-right shunt due to ostium primum atrial septal defect; mild tricuspidal and minimal mitral regurgitations with cleft of the anterior mitral valve leaflet; mild enlargement of the right cardiac chamber with pulmonary artery pressure of 40 mmHg. Chest X-ray showed widespread bronchial wall thickening and enlargement of the cardiac silhouette (Fig. 2).

The absence of other causes for pericardial effusion and the history of recent flu-like syndrome rose the suspicion of a viral etiology; therefore, laboratory evaluation identified the presence of influenza B virus on molecular assay of tracheal aspirate. Antiviral therapy with oseltamivir 75 mg twice a day for 5 days was added to ongoing treatment with metilprednisolone 30 mg (0.5 mg/kg/day) and colchicine 0.5 mg/day therapy [7].

The patient showed a progressive clinical and CRP improvement with disappearance of the pericardial effusion on 15-days echocardiographic follow-up.

The patient was discharged in good medical conditions with 1-month prednisone 30 mg/day followed by dose reduction of 2.5 mg every 5 days and 3-months colchicine 0.5 mg/day therapy [7].

At 1-month follow-up, patient was asymptomatic with normal physical examination, CRP 1.1 mg/L, and echocardiogram showed mild pericardial detachment on right atrium without sign of compression (Fig. 1b). At 6-month follow-up, clinical and echocardiographic features was preserved, with complete normalization of inflammatory markers (CRP 0,26 mg/L) even when treatment was suspended.

## Discussion

Influenza is a viral infection caused by Influenza viruses belonging to the *Orthomyxoviridae* family. They are divided in Influenza A and B virus −responsible for seasonal epidemics with 3–5 million severe cases and about 300,000 deaths per year on the world- and Influenza C virus -causing, in general, mild disease- [8]. Specifically, Influenza B virus presents two distinct subtypes -Victoria and Yamagata- circulating in humans and whose transmission can occur through fine particles (aerosol), droplet nuclei, and contact. Seasonal influenza may present with both asymptomatic and fulminant manifestations, depending on the host and virus characteristics [9]. Upper respiratory tract symptoms (nose, throat, and bronchi) are the most frequent. Pulmonary (primary viral or secondary bacterial pneumonia, exacerbations of underlying chronic lung disease) and non-pulmonary (myositis, rhabdomyolysis, renal failure, myo-pericarditis, exacerbation of coronary artery disease or heart failure, Reye syndrome, encephalomyelitis, transverse myelitis, Guillain-Barrè syndrome, aseptic meningitis, and encephalitis) complications are rare [10, 11] . Mortality rate is similar for Influenza A and B virus (16% and 10% respectively) [12].

Once the clinical suspicion appears (clinical diagnosis ranges from 29% to 80%) [13], influenza testing should be performed on nasopharyngeal samples or tracheal aspirate during the infection peak [14]. If the diagnosis is confirmed, oseltamivir 75 mg twice a day therapy must be administered for 5 days in high-risk patients within 48 h after symptom onset, in high-risk outpatients without disease improvement or positive influenza test after 48 h of symptom onset, in individuals with symptoms onset > 48 h before presentation with persisting moderate-to-severe illness, in patients requiring hospitalization [15]. Resistance to antiviral therapy has not been described for Influenza B virus infections, while occurs respectively in 27% and 3% in Influenza A H1N1 and H3N2 infection [16].

Because of its high incidence, clinical manifestation and severe complications of Influenza B virus infection are generally undervalued and rarely reported. In particular, cardio-pulmonary complications are responsible for 0.08% US hospitalizations and several post-mortem evaluations of fatal Influenza B virus infection cases highlighted myocardial damage [17]. Asymptomatic cardiac involvement occurs in 0–53% of cases [18, 19]. Kallen et al. highlighted presence of ECG alterations in about 50% of patients with Influenza [20], while presence of risk factors (age, pregnancy, genetic susceptibility, immunocompromised state, and medical comorbidity) are associated with high morbidity and mortality, mostly for coronary artery disease [21]. In our patient, a large pericardial effusion caused by Influenza B infection was diagnosed. The improvement was reached introducing neuraminidase (oseltamivir) because the patient was unresponsive to NSAID and colchicine and poor responsive to the following association with metilprednisolone. Interestingly, etiological diagnosis allowed specific therapy administration, making pericardiocentesis superfluous, risk of recurrence lower, and patient management easier.

Current literature shows some cases of myopericarditis from influenza B virus infection [22]; to our knowledge, this is the first case report of reactive pericarditis without myocardial involvement in adult due to Influenza B virus infection.

Currently, trisomy 21 is not included in the Groups at risk of influenza complications, and has no indication of primary prophylaxis [23], despite trisomy 21 was strongly associated with pericardial effusion for infectious diseases -usually viral infections-.

Congenital heart disease with pulmonary hyperflow (defect affecting up to 40% of the patients with Down syndrome) [24], such as in our patient, lead to pulmonary hypertension. Secondary pulmonary hypertension progressively leads to remodelling and dysfunction of pulmonary vessels (causing right ventricular failure) with reduced lymphatic clearance and pericardial effusion formation [25].

Specific molecular assay for Influenza B virus in nasopharyngeal swab or tracheal aspirate should be performed by clinicians during diagnostic work-up for pericarditis. On the other hand, pericarditis should be considered as a potential complication of flu-like syndrome, especially in presence of hemodynamic or respiratory failure.

Although specific antiviral along with standard therapy administration result in healing, vaccination represents the best preventive method for both adults and children with effectiveness rates of 50–60% [13, 26, 27], mostly in high-risk patients (> 65 years, young children, presence of comorbidities, immunocompromised patients) [28].

## Conclusion

Especially in autumn or winter periods, clinicians should include Influenza B virus infection among possible causes of pericarditis and large pericardial effusion. A prompt diagnostic evaluation and specific therapy administration will improve patient prognosis.

In this particular case report, history of flu-like syndrome after influenza vaccination and presence of worsening cardio-pulmonary symptoms despite typical therapy in patient with evidence of pericardial effusion rose the suspicion of a viral etiology.

To our knowledge, this is the first case report of influenza B virus infection complicated by massive and life-threatening pericardial effusion suggesting that vaccination could avoid the onset of a complicated infection. Therefore, patients with Down syndrome could be considered in the Groups at risk of influenza complications and benefit from primary prophylaxis.

## Abbreviations

CRP: C reactive protein
CT: Computed tomography
ECG: Electrocardiogram
NSAIDs: Nonsteroidal anti-inflammatory drugs
